# Received Signal Strength Database Interpolation by Kriging for a Wi-Fi Indoor Positioning System

**DOI:** 10.3390/s150921377

**Published:** 2015-08-28

**Authors:** Shau-Shiun Jan, Shuo-Ju Yeh, Ya-Wen Liu

**Affiliations:** Department of Aeronautics and Astronautics, National Cheng Kung University, Tainan 70101, Taiwan; E-Mails: P48981162@mail.ncku.edu.tw (S.-J.Y.); Q46024012@mail.ncku.edu.tw (Y.-W.L.)

**Keywords:** kriging, received signal strength database, fingerprinting, IEEE 802.11 (Wi-Fi), indoor positioning system

## Abstract

The main approach for a Wi-Fi indoor positioning system is based on the received signal strength (RSS) measurements, and the fingerprinting method is utilized to determine the user position by matching the RSS values with the pre-surveyed RSS database. To build a RSS fingerprint database is essential for an RSS based indoor positioning system, and building such a RSS fingerprint database requires lots of time and effort. As the range of the indoor environment becomes larger, labor is increased. To provide better indoor positioning services and to reduce the labor required for the establishment of the positioning system at the same time, an indoor positioning system with an appropriate spatial interpolation method is needed. In addition, the advantage of the RSS approach is that the signal strength decays as the transmission distance increases, and this signal propagation characteristic is applied to an interpolated database with the Kriging algorithm in this paper. Using the distribution of reference points (RPs) at measured points, the signal propagation model of the Wi-Fi access point (AP) in the building can be built and expressed as a function. The function, as the spatial structure of the environment, can create the RSS database quickly in different indoor environments. Thus, in this paper, a Wi-Fi indoor positioning system based on the Kriging fingerprinting method is developed. As shown in the experiment results, with a 72.2% probability, the error of the extended RSS database with Kriging is less than 3 dBm compared to the surveyed RSS database. Importantly, the positioning error of the developed Wi-Fi indoor positioning system with Kriging is reduced by 17.9% in average than that without Kriging.

## 1. Introduction

Today, various positioning technologies are used in many applications, and the users use several kinds of devices to get highly accurate location information. For example, a global positioning system (GPS) works better when the device has a clear line of sight to the sky. However, it is not suitable for indoor environments due to the fact that the GPS signal is blocked by the walls of the building. In order to provide positioning services in all environments, an indoor positioning system is developed. The indoor positioning must deal with complicated indoor environments and difficulties including, but not limited to, the multipath effect and noise interference. Additionally, the scale of an indoor environment is usually smaller than that of an outdoor environment. Thus, the anticipated positioning error for an indoor environment would be smaller than that for an outdoor environment. To improve indoor positioning accuracy and reliability, a wireless indoor positioning system is used. With the rapidly gaining popularity of Wi-Fi (IEEE 802.11)-enabled devices indoors, the access point (AP) is commonly used as a wireless device in buildings. Wi-Fi is not specifically designed for indoor positioning, but its radio signals can be employed in location estimation [1]. There have been a number of studies on Wi-Fi-based indoor positioning, including signal strength and signal time-of-flight [1,2,3,4]. The advantage of Wi-Fi radio signals is that they are more robust, accurate, and cost-effective in indoor environments [3]. Thus, an IEEE 802.11 indoor positioning system is developed in this paper. The main approach of the developed indoor positioning system is based on the received signal strength (RSS) observations.

For RSS based techniques, the positioning system requires no additional special hardware. The indoor positioning system can be implemented by exploiting the RSS values measured in any device equipped with Wi-Fi functions, such as computers and smartphones [1]. The common positioning approach based on RSS is the fingerprinting approach. The RSS based fingerprinting approach includes two steps, namely the training (calibration) stage and the positioning (verification) stage. The training stage is to build the RSS database for the indoor environment of interest, and then the positioning stage determines the user location by matching the actual RSS measurement with the RSS database obtained from the training stage. Importantly, the performance of the fingerprinting approach is determined on the establishment of the fingerprint database size. The smaller the granularity, the better estimation of the user position [2]. That is, it takes more time to measure more reference points (RPs) necessary to extend the database size. To solve this problem, many interpolation methods were proposed to create or extend the RSS database for the training (calibration) stage. A probabilistic approach to generate a Wi-Fi fingerprint database was proposed in [5] and a sparsity-based recovery approach was proposed in [6] to recover the missing RSS samples in the database. In [7] a RSSI geography weighted regression method is proposed to take advantage of features of the signal attenuation model. In this work, an efficient and effective database interpolation method, the Kriging algorithm [8] is used. The Kriging algorithm is one of the spatial interpolation methods and the key concept is to use observations to generate a spatial structure [9,10]. This spatial structure can be characterized by a semivariogram, which is a tool used to quantify spatial correlations between observations [2]. According to our previous ZigBee (IEEE 802.15.4) experimental results shown in [9,11,12], the Kriging algorithm is applied to yield a fingerprint database for an indoor positioning system and is able to maintain good positioning performance with a small number of RPs. However, in the experiments the ZigBee transmitters were installed at the pre-arranged locations to gain the optimal user positioning performance, and a regular grid formation over the indoor environment was used for the pre-arranged locations of the transmitters.

To construct on IEEE 802.11 indoor positioning system that focuses on RSS database generation for the fingerprinting approach, the basic RSS database consists of an appropriately small number of measured RPs and is applied to create the semivariograms of the 9 APs in the building under investigation. The nine APs are distributed over the department building, and the radio frequency band is set at 2.4 GHz. Moreover, the weakly-sensed APs which are located further away or on other floors are considered to obtain additional environmental information [1]. Instead of measuring RSS values at more unobserved locations, the RSS values used to extend the basic database are produced by the Kriging algorithm using a linear trend to increase the efficiency and reduce the workload. Several experiments for different size databases based on a small number of measured RPs in the database are explained, and the influences of the size of the extended database under different conditions on the performance of the positioning results are also evaluated.

The rest of this paper is organized as follows. Section 2 explains the fingerprinting approach and the Kriging algorithm for the developed IEEE 802.11 indoor positioning system. Section 3 describes the experiment setup including the hardware and software architectures, and the experiment results are analyzed and discussed in this section as well. Finally, Section 4 presents the conclusions and suggestions for future work.

## 2. IEEE 802.11 Indoor Positioning Systems

**Figure 1:**
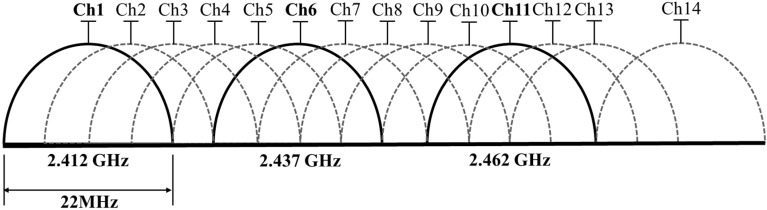
The 802.11b channels in the 2.4 GHz bands.

The IEEE 802.11, called Wi-Fi (wireless fidelity), is a wireless networking technology. The commonly used protocols are IEEE 802.11b/g/n and transmitted by APs. The IEEE 802.11b/g uses 2.4 GHz bands. The IEEE 802.11n uses both 2.4 GHz bands and 5 GHz bands. In this paper, IEEE 802.11b is selected as the communicating protocol. One of the considerations is that 2.4 GHz network covers a larger range than the 5 GHz network [13], and the other is that the APs in the building under consideration commonly uses IEEE 802.11b to send packets. A packet is defined as a piece of communicating information transmitted from a device based on the IEEE 802 series standards. In the case of the IEEE 802.11b, the non-overlapping channels are 1, 6, and 11, as shown as Figure 1, and it is more possible to increase the available performance capacity of the network in these channels [14]. As a result, the non-overlapping channels are used for the Wi-Fi indoor positioning system developed in this work.

The Wi-Fi AP only sends packets in a specific channel once. Thus, the user can only listen to one channel at a time. To discover all of the APs in an environment, the user has to scan each channel. In addition, the MAC address (basic service set identification, BSSID) and network name (service set identification, SSID) are included in the capture packets as the identification of each AP [15]. There are two scanning techniques, namely passive scanning and active scanning [16]. Passive scanning is a type of scanning where the AP broadcasts beacon frames itself to tell users of its existence, and the user just has to receive. In contrast, active scanning is that the user has to send a probe request to ask the AP if it exists or not. When the AP receives the request, it sends a probe response back to the user. In this paper, the measurements are obtained by capturing packets from APs using the passive scanning method. For the fingerprinting approach, RSS is used as the measurement based on the IEEE 802.11. The RSS is estimated with the network analysis software by capturing the broadcast packets sent from the APs. Using different software, the RSS value is different.

### 2.1. The Fingerprinting Approach

**Figure 2:**
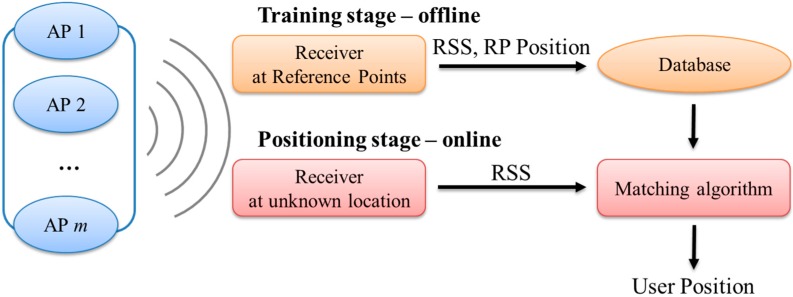
Process for the fingerprinting approach.

The fingerprinting approach consists of two stages: the training (calibration) stage and the positioning (verification) stage [9,11]. The training stage is intended to build up the database. First, an appropriate number of RPs are selected. The number of RPs determines the position granularity. Then, the RSS values are for the signals transmitted from APs that are measured at all RPs, and the measurements are recorded as fingerprints in the database. In the positioning stage, the user measures the RSS of APs at an unknown location. The measurements are considered to be a location tag. The user position can be determined after matching the measurements to the fingerprints in the database. The process for the fingerprinting approach is illustrated in Figure 2. A matching algorithm is the dominant factor related to the accuracy of the fingerprinting method. Other important factors are the RSS database generation and its size, and these factors are investigated in this work.

In this paper, the matching algorithm shown in Figure 2 is the K-weighted nearest neighbors (KWNN) algorithm. The KWNN algorithm is used to calculate the minimum distance between the RSS measurement and the data pre-recorded in the database and is used to select several RPs that are closely matched to the RSS measurement [9,17]. Then, the points selected are used to determine the positioning results. The K of the KWNN is the total number of selected points. The algorithm selects more than one point in order to reduce unexpected factors (e.g., human movement or indoor furniture will cause more signal reflection, for example, and the RSS measurement therefore will have different values with the signal propagation at the same location). To enhance the reliability of the positioning results, a greater number of selected points will reduce the error caused by irregular points. The minimum distance is calculated using Equation (1).

(1) $$d_{j}=\frac{1}{N_{S}}{\left(\sum_{i=1}^{N_{S}}{\left|rss\left(pos,i\right)-RSS\left(j,i\right)\right|}^{p}\right)}^{\frac{1}{p}}$$

where rss(pos,i) is the received signal strength of the *i_th_* AP at user position *pos*; RSS(j,i) is the received signal strength of the *i_th_* AP at the *j_th_* RP in the database, and *N_S_* is the total number of APs. According to [2], when 1/*p* is close to 1, a smaller distance means that the difference in the RSS between the RP and the user position is smaller. That is, the user position is more similar to the RP. Thus, the RP with a smaller distance has a larger weighting in the positioning results. The weighting value of each RP is determined by Equation (2).

(2) $$W_{j}=\frac{1/d_{j}}{\sum_{j=1}^{K}1/d_{j}}$$

where *K* is the total number of the selected RPs. The user position is estimated by Equation (3):

(3) $$\left(X,Y\right)=\sum_{j=1}^{K}\left[W_{j}\times \left(X_{j},Y_{j}\right)\right]$$

where $\left(X_{j},Y_{j}\right)$ is the coordinate of the selected RP*_j_*.

### 2.2. Kriging Algorithm

**Figure 3:**
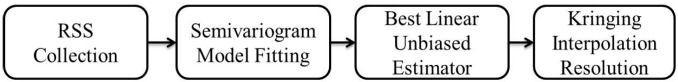
Flow chart of the Kriging algorithm.

The Kriging algorithm is utilized to reduce the training effort and to enhance the positioning performance. The Kriging algorithm predicts a value at an unobserved location based on known data. The purpose is to create a spatial structure which can be represented by a mathematical function to provide continuous values. The assumption of the Kriging algorithm is that a spatial random variable is spatially dependent, that is, the nearby points are more similar than those that are far away. In this paper, the spatial random variable is the RSS of the IEEE 802.11. The flowchart of the Kriging algorithm is depicted in Figure 3:

The semivariance is an important concept in the Kriging interpolation, and it describes the spatial correlations between observations [8]. Assume that the spatial random variable is stationary; that is, spatial random variables have a correlation between relative locations and a non-correlation between absolute locations [10]. The theoretical semivariance *γ* can be defined as Equation (4). For estimating the Kriging predictor, the spatial covariance function *Cov* is used. In the case of covariance stationarity, the relationship of *γ* and *Cov* is shown as Equation (5).

(4) $$\gamma \left(h\right)=\frac{1}{2}Var\left[z\left(u_{i}\right)-z\left(u_{j}\right)\right]$$

**Figure 4:**
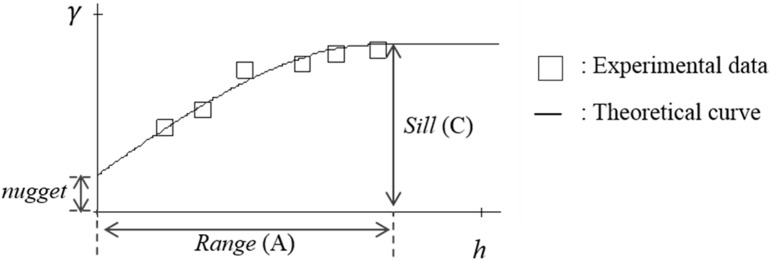
Theoretical semivariogram and experimental semivariogram.

(5) γ(h)=Cov(0)−Cov(h)

where *h* is the distance between points $u_{i}$ and $u_{j}$; *Var* is the variance; z(u) is the RSS at point *u*. For real data, the experimental semivariance $\hat{\gamma }\left(h\right)$ is mathematically estimated by Equation (6):

(6) $$\hat{\gamma }\left(h\right)=\frac{1}{2N\left(h\right)}{\sum_{N\left(h\right)}\left[z\left(u_{i}\right)-z\left(u_{j}\right)\right]}^{2}$$

where N(h) is the number of pairs of sample points separated by *h*. By Equation (6), an experimental semivariogram can be built. Thus, the experimental semivariogram and the theoretical semivariogram can both be drawn on a plot of *γ* against *h*, as shown in Figure 4.

In Figure 4, when the distance increases to a specific range, the *γ* is stable. The range is called the range *A* and the corresponding *γ* is the sill *C* [9]. Range *A* is the influence range in the experiment, and sill *C* is the critical semivariogram in the influence range. The parameters *A* and *C* are used to determine the theoretical model. In this paper, there are three theoretical semivariogram models, namely Spherical model, Exponential model, and Gaussian model, which are shown as Equations (7)–(9), respectively:

(7) $$\gamma \left(h\right)=\{\begin{array}{cc} C\left[\frac{3h}{2A}-\frac{1}{2}{\left(\frac{h}{A}\right)}^{3}\right] & for\;h\leq A\\ C & for\;h>A \end{array}$$

(8) $$\gamma \left(h\right)=C\left[1-\exp\left(-\frac{h}{A}\right)\right]$$

(9) $$\gamma \left(h\right)=C\left[1-\exp\left(-\frac{h^{2}}{A^{2}}\right)\right]$$

The Kriging system is a geostatistical method for optimal spatial prediction at unobserved locations [10,18]. The main idea of the Kriging algorithm is to use known measurements to formulate a function for yielding an unbiased estimation with the minimum error variance at unobserved locations, and the function is determined by the semivariogram. The Kriging estimator satisfies the best linear unbiased estimate (BLUE). All Kriging estimators are variants of the basic linear regression estimator $\hat{z}\left(u_{0}\right)$ defined as Equation (10):

(10) $$\hat{z}\left(u_{0}\right)=\sum_{i=1}^{n}\lambda_{i}z\left(u_{i}\right)$$

where *u*_0_ is the unobserved location; *z*(*u_i_*) is the known measurement at point *u_i_*; *n* is the total number of the known measurements, and *λ_i_* is Kriging weight of *z*(*u_i_*). The *λ_i_* is estimated by Equation (11):

(11) $$\left[\begin{array}{c} \lambda_{1}\\ \vdots \\ \lambda_{n} \end{array}\right]={\left[\begin{array}{ccc} \gamma_{11} & \cdots & \gamma_{1n}\\ \vdots & \ddots & \vdots \\ \gamma_{n1} & \cdots & \gamma_{nn} \end{array}\right]}^{-1}\left[\begin{array}{c} \gamma_{10}\\ \vdots \\ \gamma_{n0} \end{array}\right]$$

where *γ_ij_* is the semivariogram between *z*(*u_i_*) and *z*(*u_j_*), and *γ_i0_* is the semivariogram between observed value *z*(*u_i_*) and the unobserved value *z*(*u*_0_). Then, Equation (10) satisfies the unbiased constraint as shown in Equation (12):

(12) $$E{\left[\hat{z}\left(u_{0}\right)-z\left(u_{0}\right)\right]}^{2}=0$$

and Equation (10) has the minimum variance as Equation (13):

(13) $$min\left\{Var\left[\hat{z}\left(u_{0}\right)-z\left(u_{0}\right)\right]\right\}$$

## 3. Experimental Database and Positioning Results

**Figure 5:**
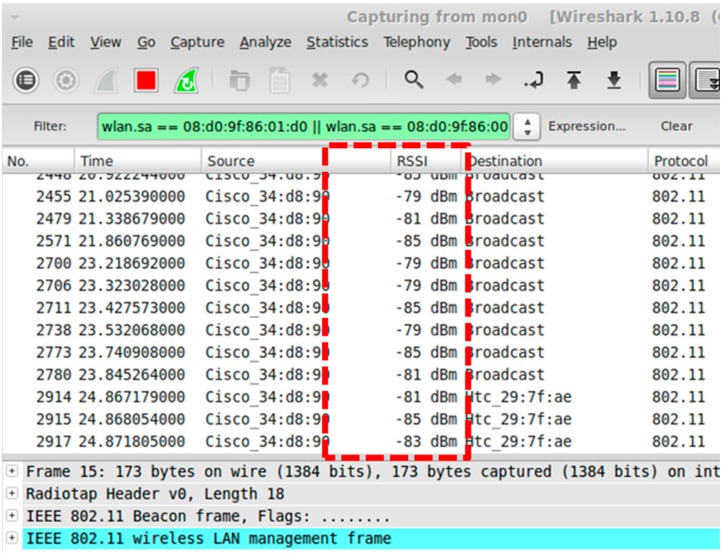
A Wireshark screenshot.

The Department of Aeronautics and Astronautics building at National Cheng Kung University in Taiwan is used as an example to demonstrate the implementation of the IEEE 802.11-based indoor positioning system. The IEEE 802.11-based wireless APs in the building under consideration serve as the transmitters. The receiver of the RSS measurements consists of a laptop and a wireless network card. The signal processing software is Wireshark, which is a network analysis tool [19], and it is used to capture network packets and provides information, including reference time, signal source, and received signal strength indication (RSSI). Figure 5 is a screenshot of the captured packets with the Wireshark tool.

**Figure 6:**
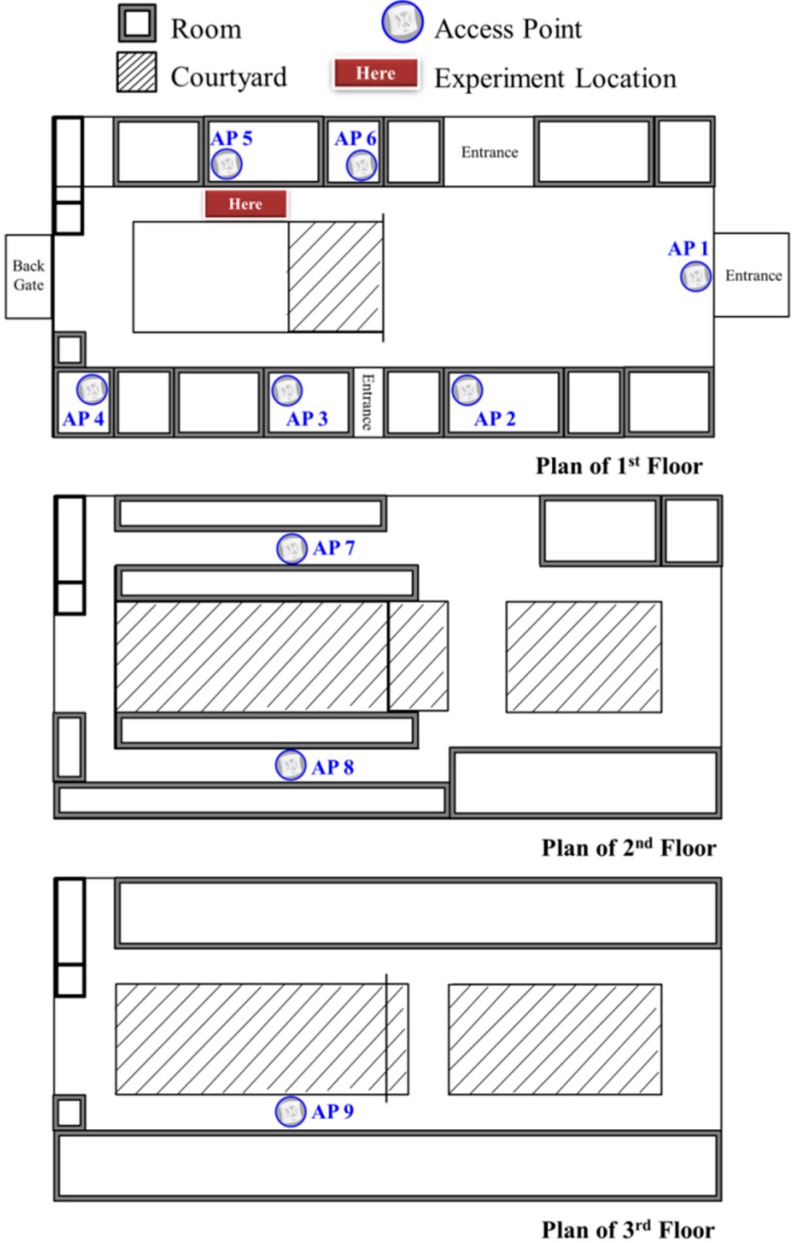
The distribution of APs and the experiment location in the building.

The first floor of the building is selected as the experiment location. The locations of the experimental points are in the corridor. We choose 9 APs to build the RSS database. The setup of the experiment is depicted in Figure 6. Figure 6 is a simple plan of the first to third floors of the department building, and it shows the relative positions between the experimental field and the 9 APs. There are six APs on the first floor, two APs on the second floor, and one AP on the third floor. AP 1 is in the lobby, and AP 9 is in the corridor. AP 7 and AP 8 are in the corridor between the offices, and the others are inside the classrooms.

**Figure 7:**
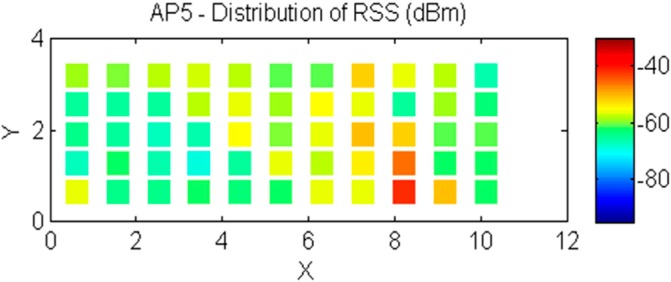
The distribution of RSS values for AP 5.

As indicated in Figure 7, we divide the experimental field into 55 points. We visit the RPs in sequence to collect the RSS measurements transmitted from the 9 APs in the building and record the RSS measurements at each point in the experimental field. Figure 7 shows the distribution of RSS values for AP 5. AP 5 has a stronger distribution of RSS values than that of the other APs because AP 5 is the nearest AP in the experimental field.

**Figure 8:**
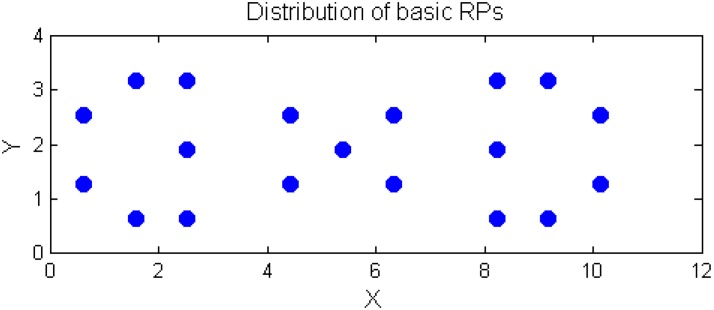
The distribution of the basic 19 RPs for both experiments for the fitting model and the fingerprinting approach.

The 19 points of the 55 points we divided in the experimental field are selected as the basic RPs, as shown in Figure 8. This special distribution of RPs is used to consider the irregular RSS measurements at some points. In addition, the 19 RPs are utilized to build the spatial model for the experimental location.

**Figure 9:**
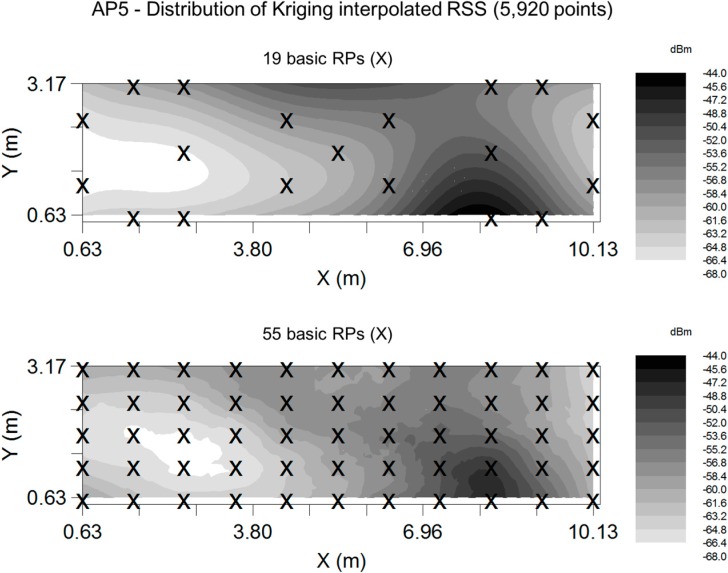
The Kriging RSS map for the AP 5.

For the RSS-based positioning system, this paper utilizes the fingerprinting approach with the KWNN algorithm. In addition, the Kriging algorithm are applied to generate training points and extend the RSS database for the purpose of positioning. The number of measured points is 55 in the experimental location. We use AP 5 to evaluate the influence of different selections of RPs on the Kriging interpolated RSSs. Figure 9 shows the Kriging RSS maps of the experimental location based on two different RP selections which are 19 and 55 points. As shown in Figure 9, the distribution of RSS based on 19 RPs (top plot) is similar to the one based on 55 RPs (bottom plot). Since the goal of this paper is to reduce the measuring effort on real data at the training (calibration) stage, 19 basic RPs of the 55 measured points are selected to estimate semivariograms and obtain influence range *A* and sill *C*.

**Figure 10:**
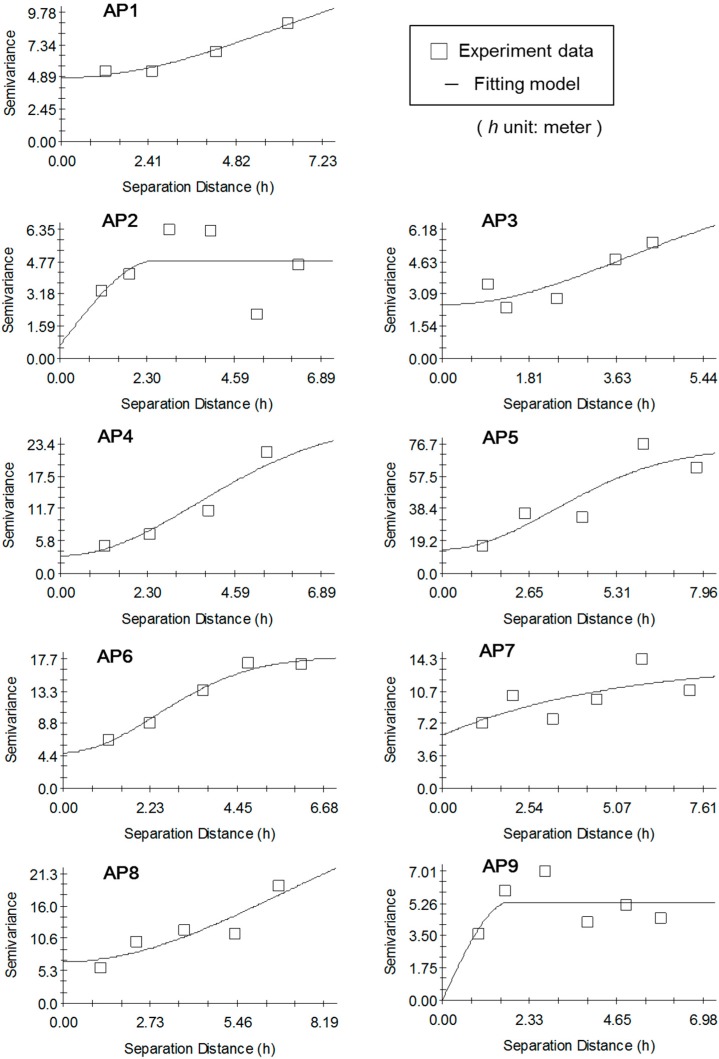
The fitting model and experimental data for the 9 Aps.

The parameters of the three fitting models based on 19 RPs are given in Table 1. The criterion for fitting model choosing is the residual sum of squares (SS) defined in [20]. When the residual SS is smaller, the fitting model is better. The better fitting models for the 9 APs are shown as Figure 10. For different APs, the points with irregular RSS measurements appear at different points although they are in the same experimental field.

**Table 1:** The parameters based on 19 RPs for the theoretical models.

	AP 1	AP 2	AP 3	AP 4	AP 5	AP 6	AP 7	AP 8	AP 9
Range *A* (m)	14.99	2.55	9.19	8.33	8.38	5.87	15.36	16.54	1.81
Sill *C* (m)	14.69	4.84	8.13	27.29	74.20	17.95	14.13	34.61	5.34
Fitting Model *	G	S	G	G	G	G	E	G	S

* “G” for Gaussian, “E” for Exponential, and “S” for Spherical.

**Figure 11:**
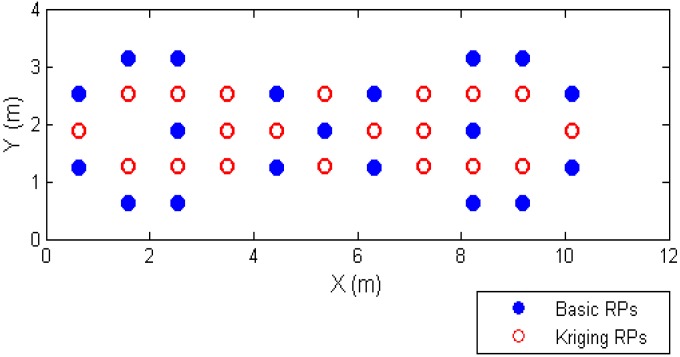
The fitting model and experimental data for the 9 Aps.

Using the 3 fitting models for each corresponding AP shown in Figure 10 with the Kriging algorithm, the RSS values can be estimated and can be compared to real measurements. There are 20 test points (red circles) given in Figure 11, and Figure 11 shows the relative positions between the test points and 19 basic RPs. In this paper, the 20 test points are randomly selected from 55 measured points, excluding the 19 basic RPs, and are used to assess the performance of the Kriging algorithm with the 9 fitting models for each corresponding AP.

**Figure 12:**
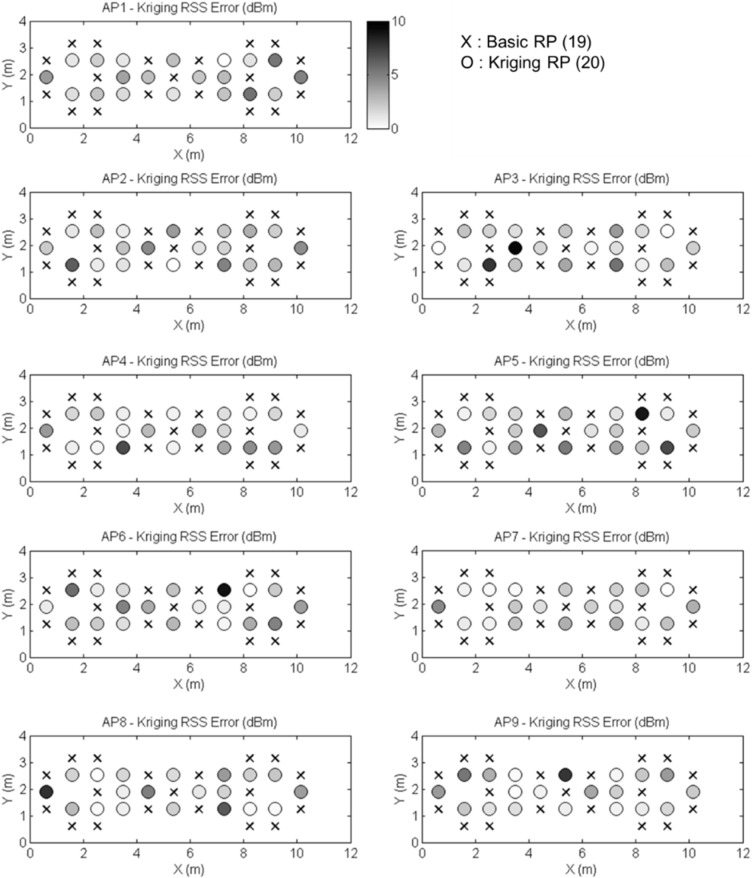
The RSS interpolation errors for 20 Kriging RPs.

The results of the interpolations of the RSS values are shown in Figure 12. The color bar indicates the error values, and the darker color means a larger error at the points. In Table 2, we calculate mean absolute error (MAE) between the truths and predictions and the standard deviation (STD). The truths of the RSS values are the real measurements. The values of MAE should be as small as possible. In the 20 test points, the 72.2% error in the Kriging interpolated values is under 3 dBm.

**Table 2:** The RSSs for the Kriging interpolated errors for 9 APs (dBm).

	AP 1	AP 2	AP 3	AP 4	AP 5	AP 6	AP 7	AP 8	AP 9
MAE (dBm)	2.49	2.57	2.58	2.13	3.09	2.69	1.77	2.31	2.20
STD (dBm)	1.51	1.62	2.46	1.79	2.33	2.18	1.20	2.16	1.95

After finishing the Kriging models for the extended database, the positioning performance using the Kinging interpolation method is evaluated. The sizes of the different databases are shown in Table 3. The 19 basic RPs are shown in Figure 8. The Case 1 uses 19 basic RPs and 20 points with real data (*i.e*., 39 basic RPs), and the other uses 19 basic RPs and 20 points with Kriging interpolation. We used Case 1 to compare the real data and the Kriging interpolated points for both experiments with the same 39 RPs as shown in Figure 11.

**Figure 13:**
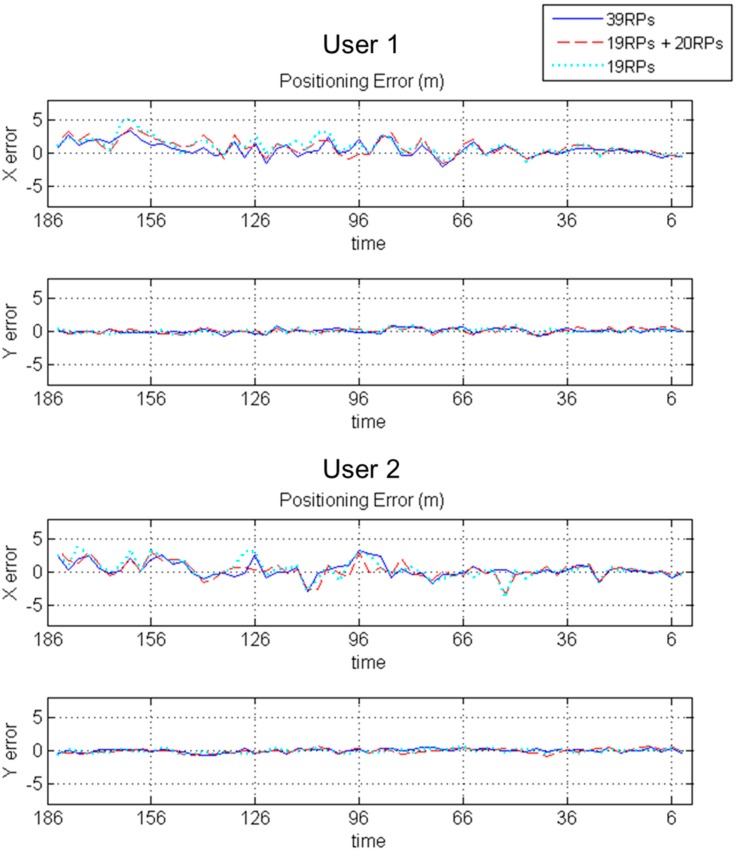
The positioning errors of 61 user points for two users.

In this work, there are two users (User 1 and User 2) moving in the same path. The user path is divided into 61 points for the purpose of measurement. The distance between each point is 15.85 cm which is a half piece of a tile on the floor. The receiving time for the data at each user point is 10 s, and the average RSS value in the 4th to the 7th s is seen as the experimental measurement. The purpose is to capture packets from the greatest number of APs and to reduce any uncertain changes in RSS measurements. For the two users, the X direction and Y direction positioning errors are given in Figure 13. The units of positioning results are in meters. The width of the Y scale in the experimental field is 2.54 m, and that of the X scale is 10.13 m. The error trend with the 19 basic RPs and 20 Kriging interpolated points is similar to that with 39 basic RPs.

**Table 3:** The RP numbers for five cases.

	Basic RPs	Kriging RPs	Total RPs
Origin	19	0	19
Case 1	39	0	39
	19	20	39
Case 2	19	38	57
Case 3	19	70	89
Case 4	19	88	107
Case 5	19	120	139

**Figure 14:**
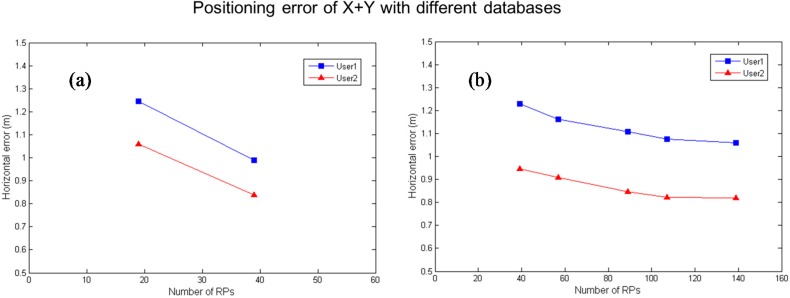
The horizontal positioning results for the two users by different Kriging interpolation points. (**a**) without Kriging database; (**b**) with Kriging database.

In Table 3, Cases 2 to 5 include more Kriging interpolation points to evaluate the possible performance gain on positioning. The horizontal positioning results for the different cases for the two users are illustrated in Figure 14. The left plot is the positioning results based on 19 RPs and 39 RPs without the Kriging interpolated database, and the right plot is the positioning results with the Kriging interpolated database. As shown in the Figure 14, a larger database size results in better positioning performance.

The data for the positioning results are given in Table 4 and Table 5. Table 4 and Table 5 show the positioning errors and the standard deviations of positioning errors using the fingerprinting approach for User 1 and User 2, respectively. In Table 4 and Table 5, the upper row of Case 1 is the positioning result with 39 basic RPs (*i.e.,* 39 real RSS data points), and the lower row of Case 1 is the positioning result with 19 basic RPs and 20 points with Kriging interpolation (*i.e.,* only 19 real RSS data points). As noted earlier, the Case 1 is used to evaluate the real RSS data and the Kriging interpolated RSS data for both experiments with the same 39 RPs as shown in Figure 11. The X+Y error represents the distance error. Then, we compare the positioning results for five cases to the original results. The errors and the standard deviations for the five cases in the X direction are smaller than those of original one. The 57, 89, 107 and 139 RPs using the Kriging algorithm have better positioning performance in the X direction than that of the original one in our experimental range. For User 1, the positioning performance is improved up to 15.5% of the error in the X direction positioning results and up to 14.9% of the error in the horizontal positioning results. For User 2, the positioning performance is improved up to 22.7% of the error in the X direction positioning results and up to 20.9% of the error in the horizontal positioning results. In the Y direction, the improvement in the results is not significant due to the fact that the X scale is 3.75 times larger than the Y scale in the experimental field. Both users exhibit similar performance. As a result, the Kriging algorithm can be applied to extend the RSS database, and the Kriging interpolation points can be used to enhance the positioning performance.

**Table 4:** The positioning performance of User 1 (Unit: m).

	X Error	Y Error	X + Y Error	X STD	Y STD
Origin (19)	1.218	0.258	1.245	1.295	0.336
Case 1 (39)	0.951	0.271	0.989	1.101	0.337
	1.191	0.308	1.230	1.242	0.366
Case 2 (57)	1.124	0.298	1.163	1.212	0.358
Case 3 (89)	1.064	0.312	1.109	1.078	0.373
Case 4 (107)	1.046	0.244	1.074	1.137	0.319
Case 5 (139)	1.029	0.247	1.058	1.076	0.347

**Table 5:** The positioning performance of User 2 (Unit: m).

	X Error	Y Error	X + Y Error	X STD	Y STD
Origin (19)	1.061	0.247	1.089	1.430	0.299
Case 1 (39)	0.839	0.236	0.871	1.153	0.287
	0.946	0.272	0.984	1.286	0.334
Case 2 (57)	0.908	0.277	0.949	1.189	0.335
Case 3 (89)	0.848	0.701	0.889	1.136	0.328
Case 4 (107)	0.823	0.267	0.865	1.097	0.333
Case 5 (139)	0.820	0.261	0.861	1.101	0.331

## 4. Conclusions

In this paper, an IEEE 802.11-based indoor positioning with the RSS based system was developed. The performance of the fingerprinting approach was determined by the size of the database. To lower the labor of the extended database, the Kriging algorithm was applied to the 9 APs existing in the building, and 19 measured points were used to build the fitting models. Comparing the interpolated database to the measured database, the mean error of the 9 APs was found to be 2.43 dBm. There is a 72.2% probability of interpolating an RSS value with an error under 3 dBm. By using five cases including different database sizes based on the Kriging interpolated points, the positioning performances in the moving direction were improved. The horizontal positioning error was reduced by 17.9%. Thus, the Kriging algorithm was applied to improve the efficiency of the training stage by adding more virtual reference points (RPs) to enhance the positioning database. The obvious improvement for positioning results will be obtained in larger scale of the experiments. Finally, the IEEE 802.11 indoor positioning system utilizing the Kriging algorithm for the database was successfully implemented in the Department of Aeronautics and Astronautics building at National Cheng Kung University.
